# Supplementary material for: Induction of territorial dominance and subordination behaviors in laboratory mice
Source: Sci Rep. 2024 Nov 19;14:28655. doi: 10.1038/s41598-024-75545-4 (PMC11577026; doi:10.1038/s41598-024-75545-4)
Supplement: Supplementary file 1 — Supplementary Figures. [file 41598_2024_75545_MOESM1_ESM.docx]

**Supplementary figures**

******

***Figure S1. Evolution of territorial behavior in CD1 mice***

Quantification of the evolution of territorial behavior differences between dominant and subordinate CD1 mice across subintervals (10 minutes) of the early and late observation periods (N = 10; Wilcoxon matched-pairs signed rank test: *p < .05, **p < .01, ***p < .001; actual p values are reported in supplementary material; mean ± SEM).

******

***Figure S2*. Evolution of territorial behavior in** **C57BL/6** **mice**

Quantification of the evolution of territorial behavior differences between dominant and subordinate C57BL/6 mice across subintervals (10 minutes) of the early and late observation periods (N = 18; Wilcoxon matched-pairs signed rank test; : *p < .05; actual p values are reported in supplementary material; mean ± SEM).

***Figure S3*. Evolution of territorial behavior in aggressive C57BL/6 mice**

(**a**) Quantification of the evolution of territorial behavior differences between dominant and subordinate aggressive C57BL/6 (pairs showing at least one attacak, N = 11) mice across subintervals (10 minutes) of the early and late observation periods (Wilcoxon matched-pairs signed rank test; mean ± SEM). (**b**) Correlation matrix between territorial behaviors within aggressive C57BL/6 mice (N = 11 pairs, only significant correlation coefficients are reported). (**c**) Comparison of PC1 difference (dominant minus subordinate) values between strains revealed that a significant difference persisted between aggressive C57BL/6 mice and the other strains (Dunn correction for pairwise comparisons, p-values for CD1 vs. C57BL/6 = .021, C57BL/6 vs. hybrids = .023; mean ± SEM).

***Figure S4.* Evolution of territorial behavior in CD1xB6 F1 hybrid mice**

Quantification of the evolution of territorial behavior differences between dominant and subordinate CD1xB6 F1 hybrid mice across subintervals (10 minutes) of the early and late observation periods (N = 18; Wilcoxon matched-pairs signed rank test: *P < .05, **P < .01, ***P < .001; mean ± SEM).


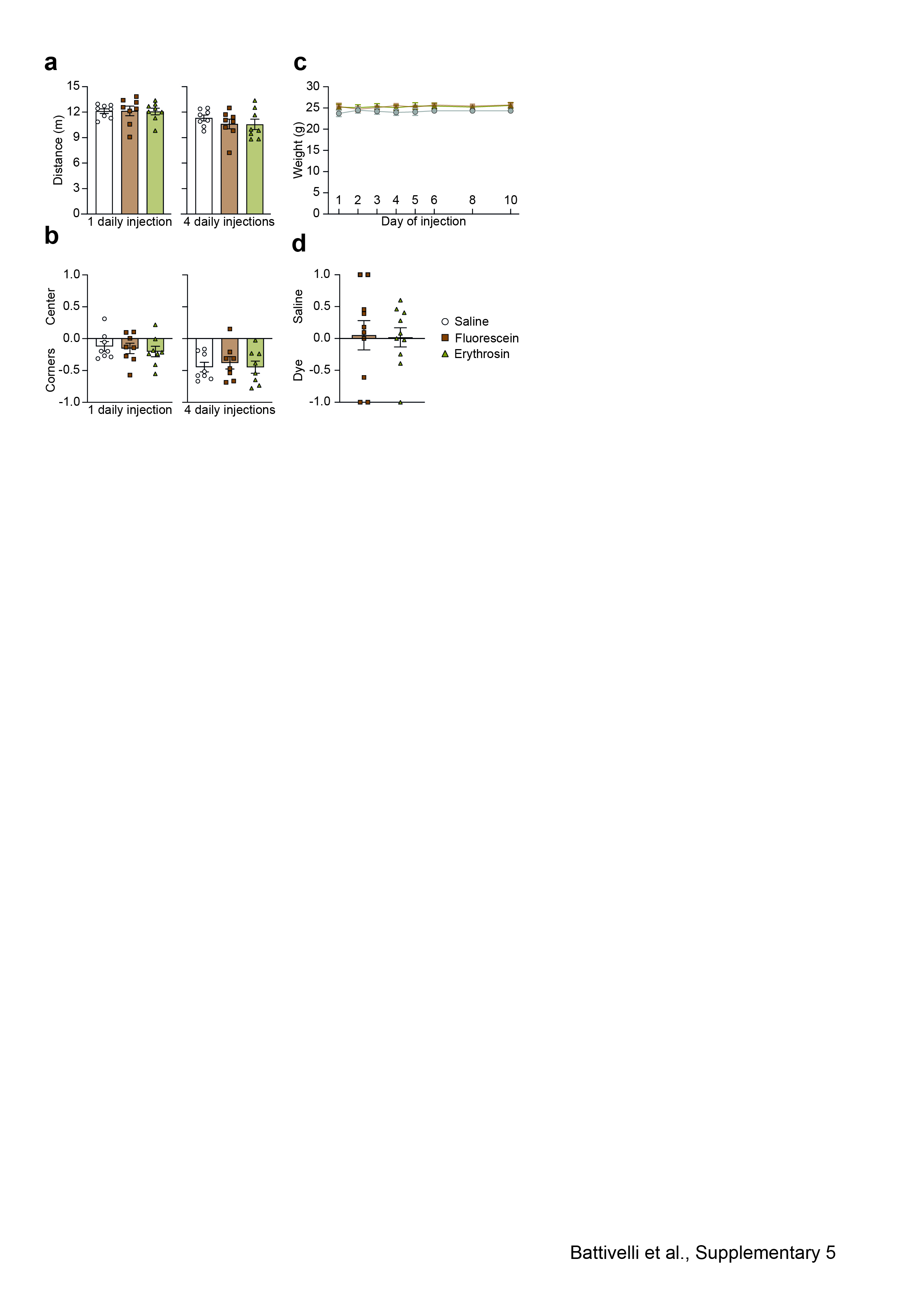


***Figure S5*. Behavioral impact of urine marking dye injection**

Treatment of C57BL/6 mice with either acute or six days injection of either fluorescein or erythrosin b was not associated with significant differences in (**a**) distance traveled (left) or (**b**) time in center vs corners (right, anxiety-like behavior index: values of 1 and -1 indicate, respectively, a preference for the center or corners of the open field with an index value of 0 indicating no preference in the open field (N = 8; Dunn correction for pairwise comparisons: all p > 0.99). (**c**) Similar chronic treatment (once daily injection for ten days) of C57BL/6 mice was not associated with significant differences in body weight (N = 8). (**d**) Odor preference for urine collected from C57BL/6 mice subjected to acute treatment was not significantly different from that expressed toward urine collected from saline treated controls (N = 10; index values of -1 and 1 indicate, respectively, a preference for the dye-containing or control urine; the index value of 0 indicates no preference; Mann-Whitney *U* test: p = 0.8; mean ± SEM).
